# Vacant Chair in the Med. Dept. of the University of the City of N. Y.

**Published:** 1847-07

**Authors:** 


*Vacant chair in the Med. Dept. of the University of the city of N. Y.*

We perceive by some of our exchanges that applications for the chair rendered vacant by the decease of the late Prof. Revere, are advertised for, until the middle of August next. By the constitution of the University, appointments are made by the Council, upon nomination by the Medical Faculty.

